# Louisville Medical Institute

**Published:** 1840-01

**Authors:** 


### LOUISVILLE MEDICAL INSTITUTE.

This institution was opened for medical instruction in the fall of 1837, Drs. *C. Caldwell, J. E. Cook, H. Miller, J. Cobb, J. B. Flint, and L. P. Yandell,* composing its Faculty, the first season; to which Dr. C. W. Short was added the next spring, and Dr. Daniel Drake in September, 1839—making in all eight Professors, namely, of the *Institutes of Medicine and Medical Jurisprudence, the Theory and Practice of Medicine, Obstetrics and the Diseases of Women and Children, Anatomy, Surgery, Chemistry and Pharmacy, Materia Medica and Medical Botany,* and *Clinical Medicine and Pathological Anatomy.* The number of students the first winter was 80; the second 120; and the number in attendance upon the present course of Lectures is 204, showing a rapidity of increase altogether unprecedented in the history of American schools of medicine. By the [illegible]nificence of the city of Louisville, a splendid and most commodious edifice has been erected for the Institute, and from the same source, it has been furnished with a large and well-selected library, chemical apparatus, &c. &c. The Marine Hospital affords to pupils the most desirable opportunities for studying disease at the bed-side, and the extensive commerce of the city opens a wide field for the study and practice of operative surgery. The peculiar advantages of Louisville as a site for a school of medicine, have long attracted the attention of medical men, and it seems to be universally conceded, that it must be the seat of one of the great schools, which the population and wealth of the Valley of the Mississippi may naturally be expected to create and sustain.

Favored by so many natural resources, and so amply furnished with all the materials and apparatus for teaching, it was to be expected that the growth of the Institute would be rapid, nor under a wise and and able administration is there reason to doubt that its prosperity will be enduring. From Kentucky it has hitherto derived a larger number of students than from any other state, but the increase from Tennessee, Alabama, Indiana, Mississippi, Illinois, and Missouri has been great. From one of those states the number, this year, is about fifty, and from fifteen to nearly thirty, from two or three of the others. In the past success and future prospects of the school there is everything to stimulate the Faculty to high and persevering exertions. Y.

